# Comprehensive analysis of correlation coefficients estimated from pooling heterogeneous microarray data

**DOI:** 10.1186/1471-2105-14-214

**Published:** 2013-07-04

**Authors:** Márcia M Almeida-de-Macedo, Nick Ransom, Yaping Feng, Jonathan Hurst, Eve Syrkin Wurtele

**Affiliations:** 1 Department of Genetics, Development and Cell Biology, Iowa State University, Ames, IA 50011, USA; 2 Current address: Syngenta Seeds Inc, 2369 330th St, Slater, IA 50244, USA

## Abstract

**Background:**

The synthesis of information across microarray studies has been performed by combining statistical results of individual studies (as in a mosaic), or by combining data from multiple studies into a large pool to be analyzed as a single data set (as in a melting pot of data). Specific issues relating to data heterogeneity across microarray studies, such as differences within and between labs or differences among experimental conditions, could lead to equivocal results in a melting pot approach.

**Results:**

We applied statistical theory to determine the specific effect of different means and heteroskedasticity across 19 groups of microarray data on the sign and magnitude of gene-to-gene Pearson correlation coefficients obtained from the pool of 19 groups. We quantified the biases of the pooled coefficients and compared them to the biases of correlations estimated by an effect-size model. Mean differences across the 19 groups were the main factor determining the magnitude and sign of the pooled coefficients, which showed largest values of bias as they approached ±1. Only heteroskedasticity across the pool of 19 groups resulted in less efficient estimations of correlations than did a classical meta-analysis approach of combining correlation coefficients. These results were corroborated by simulation studies involving either mean differences or heteroskedasticity across a pool of *N* > 2 groups.

**Conclusions:**

The combination of statistical results is best suited for synthesizing the correlation between expression profiles of a gene pair across several microarray studies.

## Background

There is a wealth of information enclosed in the massive amount of microarray data so far accumulated in public repositories. The variety of data sets generated from the assortment of experiments is a major obstacle in the path leading from these data to information. Specific issues relating to data heterogeneity across microarray studies include differences across platforms, differences within and between labs, and/or differences among experimental factors such as treatments and tissues [1,2]. Furthermore, concerns regarding integration of studies from multiple sources in general, such as variations in design, research goals, or quality of implementation, add to these issues [3,4].

Inappropriate integration of microarray data from public repositories could lead to equivocal results [5]. The “Simpson’s paradox” [6], which refers to contradictory statistical results obtained when analysis is performed within versus across groups of data [7], is an example of mishandling of data. Blyth [8] gives an example involving the analysis of 2x2 contingency tables across two groups, and Hassler and Thadewald [9] also illustrate Simpson’s paradox when correlation coefficients are estimated from a pool of two groups versus within each group. In both cases, the paradox can be explained as results are further investigated in light of the specific statistical properties of each group of data. The “ecological fallacy” happens when the correlation of aggregated variables results in a significant relationship that is due only to aggregation rather than to any real association [10] (p. 285). An early example of an ecological fallacy can be found in Gehlke and Biehl [11], whose study of grouping effects in census tract data showed that the magnitude of correlation coefficients of two variables tend to increase as the level of census tract aggregation increases. This problem was later referred to as the “modifiable areal unit problem” and further studied by Openshaw and Taylor [12].

Combining statistical results (e.g., parameter estimates, p-values) of independent studies that address similar questions has been a standard procedure in classic meta-analyses [4,13]. This approach entails analyzing each data set independently and then combining the results, as in a mosaic. Meta-analysis of microarray data has been applied in a broader context, as some works include data spanning a wide range of purposes and designs. Parmigiani *et al.*[14], in a quest for a common gene signature across multiple cancer types, developed a statistical method to identify and assess the intersection of multiple gene expression signatures across 40 published cancer-related microarray studies. On the other hand, Wirapati *et al.*[15] and Rhodes *et al.*[16] developed specific meta-analysis methods to integrate gene expression signatures of breast and lung cancer, respectively, across independent studies of microarray data. Hu *et al.*[17] and Borozan *et al.*[18] proposed methods that extend traditional effect-size models to combine information from different microarray studies as a way to evaluate or unify lists of genes differentially expressed across them.

Another approach combines data from multiple microarray studies (termed “pooled data”) in a melting pot of data and analyzes them as a single data set. Kim and Webster [19] used public databases containing microarray data and biological traits on cytoarchitectural abnormalities from the same samples of patients belonging to three groups of major mental disorders plus a control group. Their study used gene expression data measured through two array types, the Affymetrix Human Genome U133 Set A and the Affymetrix Human Genome 95av2, and the authors carried out a correlation analysis between each gene expression and the biological traits of each subject; although not fully described in the paper, it seems the correlation analysis was performed on the pooled data set from independent studies. Subsequent gene ontology (GO) [20] enrichment analysis revealed significant overrepresentation of biological processes, such as cellular metabolism, central nervous system development, cell motility, and programmed cell death, in groups of genes that were significantly correlated with biological traits. Mentzen and Wurtele [21] and Horan *et al.*[22] have created co-expression networks for *Arabidopsis thaliana* based on parameters of co-expression similarity that were estimated from a large pool of microarray data downloaded from public repositories. Mentzen and Wurtele [21] pooled data from 963 Affymetrix gene chips, distributed across 71 independent studies encompassing diverse organs, conditions, and genotypes “to quest the transcriptome in response to a wide variety of environmentally, genetically, and developmentally induced perturbations”. Horan *et al.*[22] pooled data corresponding to 1310 Affymetrix microarrays divided among 41 independent studies. Both works used cluster analysis based on Pearson correlation coefficients as a measure of similarity of gene expression profiles from *Arabidopsis*. Mentzen and Wurtele [21] analyzed data from 21,000 gene probes on the gene chip and identified clusters of co-expressed genes as regulons. Horan *et al.*[22] used clusters to identify groups of co-regulated protein of unknown function and protein of known function encoding genes from *Arabidopsis*. In both works, GO enrichment analysis showed that networks based on gene-to-gene correlations estimated from pooling data from multiple microarray studies were not random. A similar approach has been used to obtain regulon information from a human transcriptomic network derived from almost 20,000 microarrays [23]. This analysis also showed a non random functional distribution of regulons.

From a statistical standpoint, combining data from independent microarray studies into a large pool as a single set can be acceptable if data homogeneity can be ensured across studies. Yet, this condition is nearly impossible to ensure considering that significant data heterogeneity is reported even for completely replicated microarray experiments carried out by the same lab [1]. Nevertheless, it can be argued that GO enrichment implies meaningful biology and significant GO enrichment has been shown for networks created from pooled data [19,21-23]. Moreover, information gathered through a single data set analysis has led to gene function knowledge discovery [24].

The objective of this study was to perform a comprehensive analysis of Pearson correlation coefficients estimated from pooling heterogeneous groups of data (melting pot approach) in a large-scale gene expression analysis of publicly available Affymetrix microarrays and compare it to the analysis (of the same data) that combines statistical results of individual groups (mosaic approach). Our study included two specific objectives: (a) to determine the specific effect of different means and heteroskedasticity across the many groups comprising a pool of microarray data on the sign and magnitude of gene-to-gene Pearson correlation coefficients obtained from the pool of data, and (b) to quantify the extent of bias in gene-to-gene Pearson correlation coefficients obtained from a pool of heterogeneous groups of microarray data.

In the “Methods” section of this article, we describe the statistical theory that we applied to analyze the components of Pearson correlation coefficients obtained from a pool of heterogeneous microarray groups. The “Simulation study” section provides results of a study that further tests the specific effect on Pearson correlation coefficients of only mean differences, and only heteroskedasticity across *N* > 2 groups, and the validity of our methodology when groups have a small number of elements. In the section “Application to experimental microarray data” we illustrate the results predicted by both theory and simulation with data from 10 microarray experiments. At the end of this section, we provide an assessment of the bias of correlation coefficients estimated across a pool of heterogeneous groups of microarray data. We discuss our results and summarize our conclusions in the last section.

## Methods

### Dissecting components of the Pearson correlation coefficient obtained from a pool of microarray data

Hassler and Thadewald [9] developed the asymptotic formulation to quantify and explain differences between the Pearson correlation coefficient estimated from combining two heterogeneous groups into one pool and the Pearson correlation coefficients estimated within each group. They illustrated the problem with a set of measurements on height (cm) and weight (kg) reported by 184 first-year college students with a roughly even number of males and females. As the authors emphasized in their work, male and female groups were not homogeneous because “male students are taller and heavier than female students, and variation around the mean also differs between the groups”.

The generalization of Hassler and Thadewald’s [9] asymptotic analysis for *N* heterogeneous groups (refer to their original work for specifics about the asymptotic analysis) is provided in Equation 1. For the purpose of applying their theoretical work to analyze correlation coefficients obtained from a pool of microarray data, we consider *N* heterogeneous groups of gene expression data measured through microarrays. Each group of data can be described as a matrix *M*_*i*_ of *g* genes by *n*_*i*_ columns (each column of the matrix *M*_*i*_ corresponds to the expression of *g* genes measured through one microarray). We assume that expression levels of any given gene pair *xy* within each group, i.e. *x*,*y* ∈ *M*_*i*_, are bivariate random normal variables that are identically distributed with means *μ*_*xy*,*i*_ = (*μ*_*x*,*i*_,*μ*_*y*,*i*_) and variance-covariance matrix $\Sigma_{\text{xy},i}=\left(\begin{array}{cc} \sigma_{x,i}^{2} & \sigma_{\text{xy},i}\\ \sigma_{\text{xy},i} & \sigma_{y,i}^{2} \end{array}\right)$, ∀ *i* = 1,*N*. Therefore, heterogeneities across *N* groups of microarray data are characterized by *μ*_*xy*,*i*_ ≠ *μ*_*xy*,*j*_ and/or Σ_*xy*,*i*_ ≠ Σ_*xy*,*i*_, for *i* ≠ *j*.

The limit in probability of the Pearson correlation coefficient between expressions of genes *x* and *y*, *r*_*xy*_, obtained from a pool of *N* heterogeneous groups, as *n*_*i*_ → *∞*, is given by the expression in Equation 1:

(1) $$\begin{array}{l} r_{\text{xy}}\overset{p}{\to }\tau_{\text{xy}}\\ \;=\frac{\overset{N}{\underset{i=1}{\sum }}\lambda_{i}\sigma_{\text{xy},i}+\overset{N}{\underset{i=1}{\sum }}\overset{N}{\underset{j=i+1}{\sum }}\lambda_{i}\lambda_{j}(\mu_{x,i}-\mu_{x,j})(\mu_{y,i}-\mu_{y,j})}{\delta_{x}\delta y} \end{array}$$

where $\lambda_{i}=\frac{n_{i}}{\overset{N}{\underset{i=1}{\sum }}n_{i}}$ represents the weight of the number of microarrays of each group and the terms $\delta_{x}^{2}$ and $\delta_{y}^{2}$ correspond to the average of expression level variances weighted by *n*_*i*_ plus the weighted average of the square of mean differences across *N* groups for genes *x* and *y*, respectively (Equations 2 and 3). Hence, the limit in probability of gene-to-gene Pearson correlation coefficients obtained from combining heterogeneous groups of microarray data is a mixture of the weighted average of all covariances across *N* groups plus the weighted average of the cross product of the mean differences of genes *x* and *y* across *N* groups. Both terms are divided by a combination of the average of variances of genes *x* and *y* and the mean differences of genes *x* and *y* across *N* groups.

(2) $$\begin{array}{ll} \delta_{x}^{2}=\overset{N}{\underset{i=1}{\sum }}\lambda_{i}\sigma_{x,i}^{2}+\overset{N}{\underset{i=1}{\sum }}\overset{N}{\underset{j=i+1}{\sum }}\lambda_{i}\lambda_{j}{\left(\mu_{x,i}-\mu_{x,j}\right)}^{2} & \; \end{array}$$

(3) $$\begin{array}{ll} \delta_{y}^{2}=\overset{N}{\underset{i=1}{\sum }}\lambda_{i}\sigma_{y,i}^{2}+\overset{N}{\underset{i=1}{\sum }}\overset{N}{\underset{j=i+1}{\sum }}\lambda_{i}\lambda_{j}{\left(\mu_{y,i}-\mu_{y,j}\right)}^{2} & \; \end{array}$$

## Results

### Simulation study

This section presents results of a study using simulated data that had the purpose of further investigating correlation coefficients obtained from a pool of *N* groups under the following specific conditions: (a) occurrence of only mean differences across *N* > 2 groups and (b) occurrence of only heteroskedasticity across *N* > 2 groups. Our study also evaluated the validity of using estimates of the asymptotic expression given in Equation 1 to explain the components of the pooled correlation coefficient when groups comprising the pool of data have a small number of elements *n*_*i*_.

We performed simulations in R [25] for a generic gene pair *xy* following the procedure detailed in 1–4: 

1. For each simulated group *i*, *i* = 1,*N*, generate *n*_*i*_ data pairs from a multivariate normal distribution with parameters *μ*_*xy*,*i*_ and Σ_*xy*,*i*_ (we used the function mvrnorm in MASS [26]). Each simulated group is a matrix of 2 rows (genes) by *n*_*i*_ columns (number of elements *n*_*i*_).

2. Combine simulated data of *N* groups into one pool of 2 rows (genes) by $\overset{N}{\underset{i=1}{\sum }}n_{i}$ columns.

3. Obtain the Pearson correlation coefficient from the pool of data (we used the function cor in R [25]).

4. Repeat steps 1–3 above 1000 times; results are presented as averages over 1000 repetitions.

As a control, we first performed an experiment with parameters *μ*_*xy*,*i*_ = (0,0) and $\Sigma_{\text{xy},i}=\left(\begin{array}{cc} 1 & 0\\ 0 & 1 \end{array}\right)$for all groups *i* = 1,10. This simulation provided nearly zero correlation coefficients (−0.004 ≤ *r*_*xy*_ ≤ 0.003), thus reassuring us that our simulation procedure worked as expected.

### Simulation of only mean differences across a pool of *N* groups

**Figure 1:**
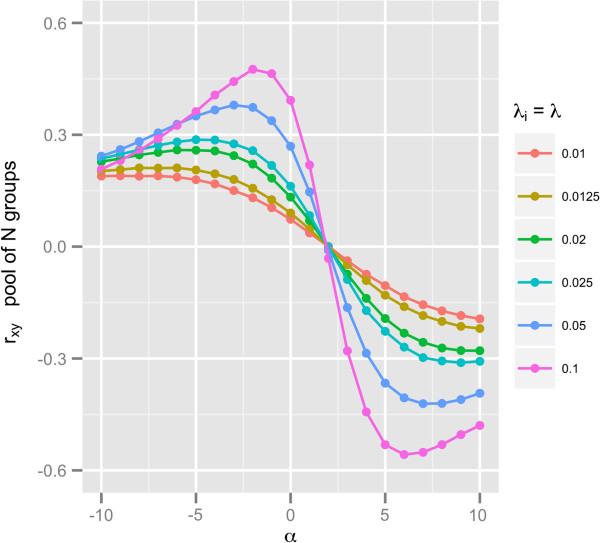
**Mean differences across a pool of** ***N* groups causes spurious correlations.** *r*_*xy*_ was obtained from combining *N* groups of simulated data; simulation parameters: *μ*_*xy*,1_ = (*α*,0), *μ*_*xy*,2_ = (0,*α*), −10 ≤ *α* ≤ 10, and *μ*_*xy*,*i*_ = (2,2) for *i* ≥ 3; *n*_*i*_ = 10, *λ*_*i*_ = *λ*, 0.01 ≤ *λ* ≤ 0.1, ∀ *i* = 1,*N*, and 10 ≤ *N* ≤ 100.

First, we analyzed the case in which heterogeneities across *N* groups of simulated data were due only to mean differences, i.e. *μ*_*xy*,*i*_ ≠ *μ*_*xy*,*j*_ for *i* ≠ *j*, but variance-covariances remained constant, i.e. Σ_*xy*,*i*_ = Σ_*xy*_. We first simulated the case of zero correlation within each group and the effect of differing means (by a parameter *α*) in only two of the *N* groups. The simulation results for the set of parameters *μ*_*xy*,1_ = (*α*,0), *μ*_*xy*,2_ = (0,*α*), −10 ≤ *α* ≤ 10, and *μ*_*xy*,*i*_ = (2,2) for *i* ≥ 3; $\Sigma_{\text{xy},i}=\left(\begin{array}{cc} 1 & 0\\ 0 & 1 \end{array}\right)$, *n*_*i*_ = 10, *λ*_*i*_ = *λ*, 0.01 ≤ *λ* ≤ 0.1, ∀ *i* = 1,*N*, and 10 ≤ *N* ≤ 100 are shown in Figure 1. The pooled correlation coefficients *r*_*xy*_ shown in Figure 1 are positive for *α* < 2, negative for *α* ≥ 2, and nearly zero for *α* = 2. Even though data pairs in each group were drawn from populations with zero correlations, *r*_*xy*_ ranged from −0.56 to 0.48. Also shown in Figure 1 is the non-linear relationship between *r*_*xy*_ and *α* as well as between *r*_*xy*_ and *λ*. Not surprisingly, coefficients *r*_*xy*_ increase as *λ* increases. The smooth curves observed in Figure 1 (as well as non-linearity of *r*_*xy*_ with *α* and *λ*) are explained by the asymptotic formulation of Equation 1 as written for the set of population parameters used in this simulation case study (Equation 4):

(4) $$\tau_{\text{xy}}=\frac{-\lambda^{2}\alpha^{2}-4\lambda (1-2\lambda )(\alpha -2)}{\lambda^{2}\alpha^{2}+\lambda (1-2\lambda ){\left(\alpha -2\right)}^{2}+4\lambda (1-2\lambda )+1}$$

Through Equation 4 one can see that $\frac{-4\lambda^{2}}{4\lambda^{2}+4\lambda (1-2\lambda )+1}\approx 0$ for *α* = 2 and 0.01 ≤ *λ* ≤ 0.1; *τ*_*xy*_ > 0 for *α* < 2 because the term −4*λ*(1−2*λ*)(*α*−2) > 0 and dominates the term −*λ*^2^*α*^2^ < 0; *τ*_*xy*_ < 0 for *α* > 2 because −4*λ*(1−2*λ*)(*α*−2) < 0 and −*λ*^2^*α*^2^ < 0.

**Figure 2:**
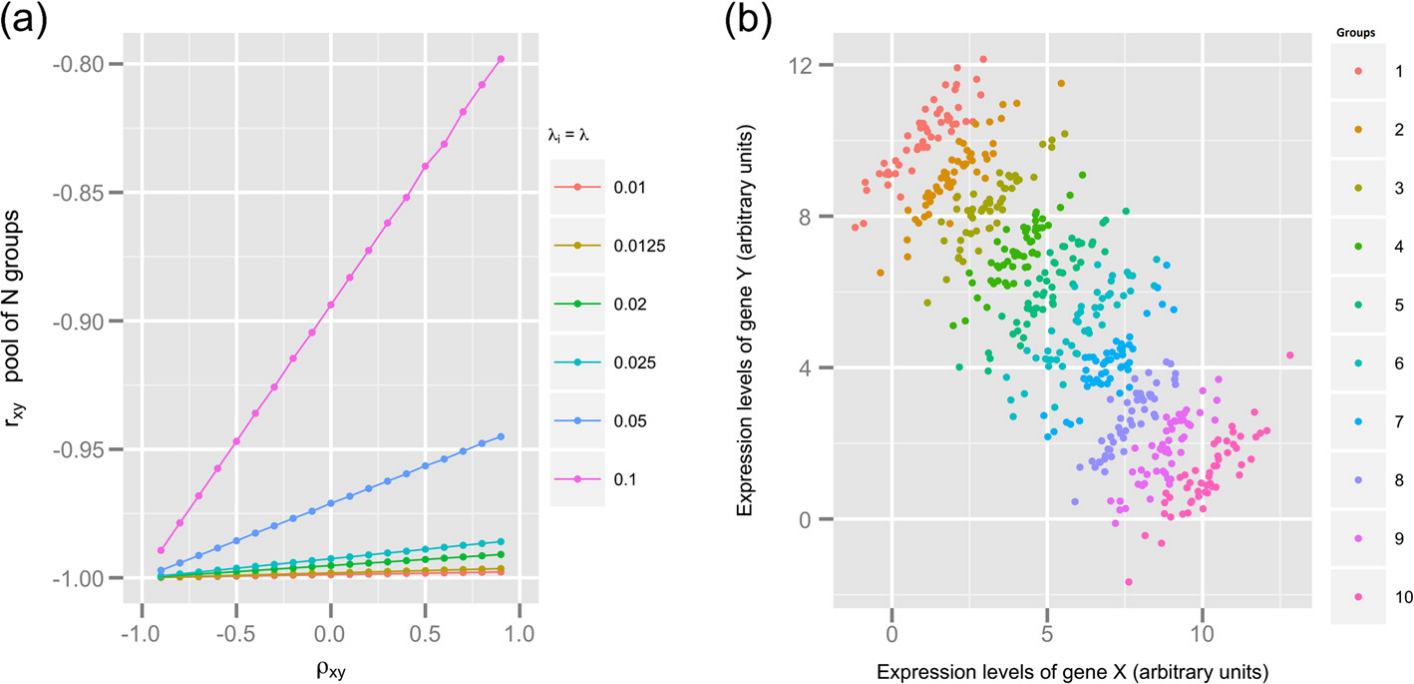
**Mean differences across a pool of** ***N* groups causes Simpson’s paradox. (a)***r*_*xy*_ was obtained from combining *N* groups of simulated data; simulation parameter *ρ*_*xy*_ is the true correlation of the pair *xy* within each group *i* = 1,*N* for . All other simulation parameters are as follows: *μ*_*xy*,*i*_ = (*i*,*N*−(*i*−1)), −0.9 ≤ *ρ*_*xy*_ ≤ 0.9, *n*_*i*_ = 10, *λ*_*i*_ = *λ*, 0.01 ≤ *λ* ≤ 0.1, for *i* = 1,*N*, and 10 ≤ *N* ≤ 100. **(b)** Scatterplot of a pair *xy* obtained with the simulation parameters: *μ*_*xy*,*i*_ = (*i*,(11−*i*)), *ρ*_*xy*_ = 0.9 and *n*_*i*_ = 50 for *i* = 1,10 groups. This plot shows clearly that even though there is a positive trend within each of the 10 groups, the trend across the pool of 10 groups is negative (Simpson’s paradox).

Secondly, we simulated the case in which the means of a gene pair *xy* differ for all *N* groups but the correlation *ρ*_*xy*_ ≠ 0 assumes the same value within each group. The simulation results for the set of parameters *μ*_*xy*,*i*_ = (*i*,*N*−(*i*−1)), $\Sigma_{\text{xy},i}=\left(\begin{array}{cc} 1 & \rho_{\text{xy}}\\ \rho_{\text{xy}} & 1 \end{array}\right)$, −0.9 ≤ *ρ*_*xy*_ ≤ 0.9, *n*_*i*_ = 10, *λ*_*i*_ = *λ*, 0.01 ≤ *λ* ≤ 0.1, for *i* = 1,*N*, and 10 ≤ *N* ≤ 100 are shown in Figure 2a. The correlation coefficients *r*_*xy*_ shown in Figure 2a are always negative, ranging from −0.99 < *r*_*xy*_ < −0.80, even when the true correlation within groups *ρ*_*xy*_ was positive. The asymptotic formulation of Equation 1 as written for the set of parameters used in this simulation case study is given in Equation 5:

(5) $$\tau_{\text{xy}}=\frac{\rho -\lambda^{2}\overset{N}{\underset{i=1}{\sum }}\overset{N}{\underset{j=i+1}{\sum }}{\left(i-j\right)}^{2}}{1+\lambda^{2}\overset{N}{\underset{i=1}{\sum }}\overset{N}{\underset{j=i+1}{\sum }}{\left(i-j\right)}^{2}}$$

Through Equation 5 one can see that *τ*_*xy*_ assumes values of nearly −1 for 10 ≤ *N* ≤ 100. A visualization of the problem considered in our second simulation case study is shown (Figure 2b) through a scatterplot of expression data of a gene pair *xy* simulated for 10 groups according to the parameters *μ*_*xy*,*i*_ = (*i*,(11−*i*)), *ρ*_*xy*_ = 0.9, and *n*_*i*_ = 50, for *i* = 1,10. As shown in Figure 2b, even though there is a positive trend between the expressions of gene *x* and gene *y* within each of the 10 groups, the trend between expression of the gene pair *xy* across the pool of all groups is negative.

### Simulation of only heteroskedasticity across a pool of *N* groups

Through simulation, we analyzed the effect of variations in $\Sigma_{\text{xy},i}=\left(\begin{array}{cc} \sigma_{x,i}^{2} & \sigma_{\text{xy},i}\\ \sigma_{\text{xy},i} & \sigma_{y,i}^{2} \end{array}\right)$among *N* groups, keeping *μ*_*xy*,*i*_ = *μ* constant. Simulation of the case where *σ*_*xy*,*i*_ = 0 but $\sigma_{x,i}^{2}\neq \sigma_{x,j}^{2}$ and $\sigma_{y,i}^{2}\neq \sigma_{y,j}^{2}$ for *i* ≠ *j* resulted in nearly zero correlation coefficients from the pool of *N* groups (−0.04 ≤ *r*_*xy*_ ≤ 0.05). This result agrees with Equation 1, which predicts zero correlation if covariances and mean differences of a gene pair across all groups are zero, even when variances differ across groups.

**Figure 3:**
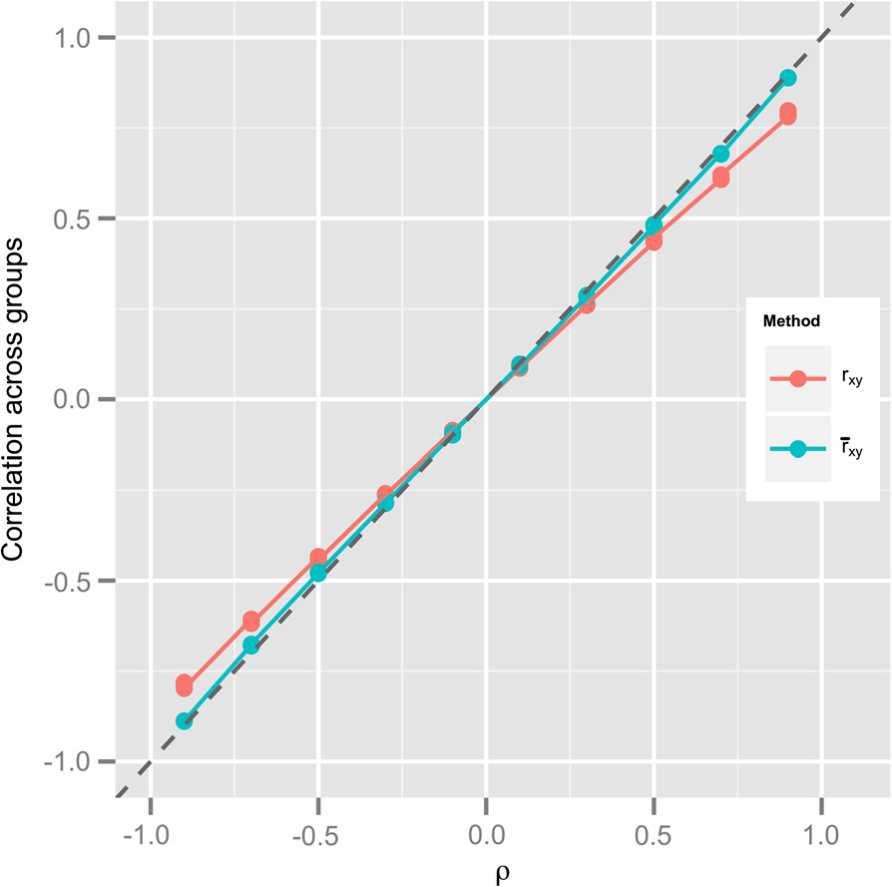
**Correlation coefficients obtained from a pool of** ***N* heteroskedastic groups.** *r*_*xy*_ was obtained from pooling *N* groups of simulated data (shown in red), and ${\bar{r}}_{\text{xy}}$ was obtained from averaging within groups correlation (shown in blue); *ρ* is the true correlation within each group. This plot shows that the error between ${\bar{r}}_{\text{xy}}$ and *ρ* is smaller than the error between *r*_*xy*_ and *ρ*. Simulation parameters: *μ*_*xy*,*i*_ = (2,2), $\sigma_{x,i}^{2}=i^{2}$, −0.9 ≤ *ρ* ≤ 0.9, *n*_*i*_ = 10, *λ*_*i*_ = *λ*, and 0.01 ≤ *λ* ≤ 0.1 for *i* = 1,*N* and 10 ≤ *N* ≤ 100.

We performed a simulation experiment in which the variance of gene *x* changes across *N* groups, but the variance of gene *y* and the correlation between genes *x* and *y* remain constant across *N* groups. Results of this experiment for the set of parameters *μ*_*xy*,*i*_ = (2,2), $\Sigma_{\text{xy},i}=\left(\begin{array}{cc} \sigma_{x,i}^{2} & \rho \sigma_{x,i}\\ \rho \sigma_{x,i} & 1 \end{array}\right)$, $\sigma_{x,i}^{2}=i^{2}$, −0.9 ≤ *ρ* ≤ 0.9, *n*_*i*_ = 10, *λ*_*i*_ = *λ*, and 0.01 ≤ *λ* ≤ 0.1 for *i* = 1,*N* and 10 ≤ *N* ≤ 100 are shown in Figure 3. The range of the pooled correlations is −0.8 ≤ *r*_*xy*_ ≤ 0.8 (shown on the y-axis of Figure 3), whereas the range of the true correlations within groups is −0.9 ≤ *ρ* ≤ 0.9 (shown on the x-axis of Figure 3). Figure 3 shows a clear linear relationship between *r*_*xy*_ and *ρ*, in which |*r*_*xy*_| < |*ρ*|. The slope between *r*_*xy*_ and *ρ*, estimated through ordinary least squares, is 0.872. Equation 6 gives the asymptotic formulation for the set of parameters used in this simulation case study:

(6) $$\tau_{\text{xy}}=\rho \frac{\bar{\sigma_{x}}}{\sqrt{\bar{\sigma_{x}^{2}}}}$$

where ${\bar{\sigma }}_{x}=\frac{\overset{N}{\underset{i=1}{\sum }}\sigma_{x,i}}{N}$ and $\bar{\sigma_{x}^{2}}=\frac{\overset{N}{\underset{i=1}{\sum }}\sigma_{x,i}^{2}}{N}$. Equation 6 also shows a linear relationship between *τ*_*xy*_ and *ρ* with a slope of $\frac{\bar{\sigma_{x}}}{\sqrt{\bar{\sigma_{x}^{2}}}}=0.872$, for *σ*_*x*,*i*_ = *i*^2^, *i* = 1,*N*, and 10 ≤ *N* ≤ 100.

There is also a linear relationship between the average of correlation coefficients within each group ${\bar{r}}_{\text{xy}}$ and *ρ* (Figure 3), in which $-0.889<{\bar{r}}_{\text{xy}}<+0.889$. In addition, as shown in Figure 3, $\left|{\bar{r}}_{\text{xy}}|>|r_{\text{xy}}\right|$. Hence, it can be easily inferred that the mean squared error between *r*_*xy*_ and *ρ* (the true correlation within each group) is greater than the mean squared error between ${\bar{r}}_{\text{xy}}$ and *ρ*. Therefore, pools of *N* simulated groups marked by only heteroskedasticity provide less efficient estimates of the correlation across groups than combining the *N* groups’ correlation coefficients into an average.

We analyzed the mean squared error between *τ*_*xy*_ and ${\hat{\tau }}_{\text{xy}}$ versus *n*_*i*_, the number of simulated elements in each group, for 10 ≤ *n*_*i*_ ≤ 100 and *N* = 20 (data shown in Additional file 1). *τ*_*xy*_ was obtained from plugging population parameters *μ*_*xy*,*i*_ and Σ_*xy*,*i*_ into Equation 1, whereas ${\hat{\tau }}_{\text{xy}}$ was based on a combination of parameters of each group ${\hat{\mu }}_{\text{xy},i}$ and ${\hat{\Sigma }}_{\text{xy},i}$ (see Equation 7). The mean squared error ranged from 0.0004 for *n*_*i*_ = 100 to 0.004 for *n*_*i*_ = 10. The correspondence between *τ*_*xy*_ and ${\hat{\tau }}_{\text{xy}}$ was good even for *n*_*i*_ = 10, a small number of simulated elements per group.

## Application to experimental microarray data

Our simulation study showed that Pearson correlation coefficients obtained from a pool of data coming from groups that have different means are explained solely by mean differences across groups. Furthermore, we showed that pooling data marked by only heteroskedasticity provides less efficient estimates of correlation coefficients than does a classical meta-analysis approach of combining correlation coefficients into an average. The following analysis of experimental microarray data illustrates the results predicted by both theory and simulation.

### Example data set

The example data set of this work includes the raw expression data from 522 Affymetrix ATH1 gene chips (cel files) from AtGenExpress [27]. Cel files are also available from The Arabidopsis Information Resourse (TAIR) [27,28]; see Table 1 for the experiment’s ID on TAIR and Additional file 2 for detailed information about treatment conditions and number of biological replicates. These data come from 10 experiments that explored the effect of 10 types of abiotic stress on RNA accumulation in shoot and root of 16 day-old *Arabidopsis thaliana* seedlings (see Table 1 for details). Experiments followed a 3-factorial design with treatment (abiotic stress, control), plant material (root, shoot or seedling), and time post-treatment as factors [27]. Seven different research groups located at different institutions across Germany performed experiments; microarray data were generated at the German Resource Center for Genome Research (RZPD) (according to experiment’s description in TAIR [28]).

**Table 1:** Description of the the example data set

**TAIR ID**	**Abiotic stress**	**Cel files**	**Plant material**	***n***_***i***_	$n_{i}^{*}$
ME00325	Cold	48	Root	12	24
			Shoot	12	24
ME00326	Genotoxic	47	Root	12	22
			Shoot	12	24
ME00327	Osmotic	48	Root	12	24
			Shoot	12	24
ME00328	Salt	48	Root	12	24
			Shoot	12	24
ME00329	UVB	56	Root	14	28
			Shoot	14	28
ME00330	Wound	56	Root	14	28
			Shoot	14	28
ME00338	Drought	56	Root	14	28
			Shoot	14	28
ME00339	Heat	67	Root	18	36
			Shoot	16	30
ME00340	Oxidative	48	Root	12	24
			Shoot	12	24
ME00345	Light	48	Seedlings	16	48

Each microarray experiment is described by its TAIR [28] id and type of abiotic stress; cel files of each experiment can be located on TAIR [28] through its id. Total number of downloaded cel files from each experiment is shown on column Cel files. The combination Abiotic stress/Plant part gives the 19 groups used for the asymptotic analysis of Pearson correlation coefficients. The column *n*_*i*_ shows number of elements in each of 19 groups comprising the large expression matrix. The column$n_{i}^{*}$ shows number of elements in each of 19 groups comprising the large matrix of residuals.

### Experimental data analysis

We imported data from cel files into the R environment [25] and processed the data with MAS5 from the open-source Bioconductor R package affy [29,30]. Following the methodology described in Horan *et al.*[22], we did not screen our example data set for quality of biological replicates, and therefore no outliers were removed. We followed this procedure because the same data from the 10 experiments of AtGenExpress [27] were also part of the larger data set used in the work by Horan *et al.*[22]. As described in the methodology of Mentzen and Wurtele [21], all data were subsequently normalized using the median absolute deviation method as performed by the function normalizeBetweenArrays (with the option “scale”) from the open-source Bioconductor R package Limma [30,31]. We obtained mean values of biological replicates after a log transformation (base 2) of the normalized expression data. Because the two treatment conditions “genotoxic stress applied to root 1 hour post-treatment” and “heat control applied to shoot 24 hours post-treatment” had data for only one biological replicate, their expression measurements were used as mean values (refer to Additional file 2 for more details). Thereafter, mean values of biological replicates were combined into one large expression matrix (pooled data) encompassing 254 columns and 22,810 rows (corresponding to probe ids/genes). All but two columns of the large expression matrix resulted from averaging data of two or three biological replicates (refer to Additional file 2 for exact number of biological replicates per treatment condition). Gene-to-gene Pearson product-moment correlation coefficients (*r*_*xy*_) were obtained from the large expression matrix (pooled data) with the R function cor. [25].

### Mean differences across a pool of microarray data

We used estimates of the asymptotic expression given in Equation 1 to examine the makeup of Pearson correlation coefficients obtained from pooling the means of biological replicates of different experimental conditions into one large expression matrix. In order to accomplish this task, we classified data in columns of the large expression matrix into 19 groups. Each group had gene expression values from either root or shoot in each of nine types of abiotic stress treatments (see Table 1 for details). We adopted this procedure because an exploratory analysis showed clear mean differences in gene levels expressed in root or shoot. The light stress experiment was for entire seedlings, and our analysis did not show mean differences that would justify further division of the data from this experiment. Each group’s name and its corresponding number of elements *n*_*i*_, for groups *i* = 1,19, are given in Table 1 (number of elements *n*_*i*_ correspond to the number of mean expression values of a gene within group *i*).

Data across 19 groups were obviously not homogeneous because each group corresponds to a combination of the type of abiotic stress and the plant material, which surely would have an effect on the total group mean of a gene expression. In addition, data within groups cannot strictly be considered homogeneous either because gene mean expression values within groups correspond to different time points post application of abiotic stress/control treatments (further details about treatment conditions inside and across groups is given in Additional file 2). Because our exploratory analysis indicated that the grand mean expression level of genes within groups seemed to dominate over means of all other treatment effects (data not shown), we considered data within groups as roughly homogeneous.

We used the procedure described in steps 1 through 4 below to make a diagnostic of *r*_*xy*_ obtained from a pool of gene expression data coming from 19 heterogeneous groups, where *μ*_*xy*,*i*_ ≠ *μ*_*xy*,*j*_ and/or Σ_*xy*,*i*_ ≠ Σ_*xy*,*j*_, for *i* ≠ *j*. 

1. For a given gene pair *xy*, obtain estimates${\hat{\mu }}_{\text{xy},i}=({\bar{x}}_{i},{\bar{y}}_{i})$ and${\hat{\Sigma }}_{\text{xy},i}=\left(\begin{array}{ll} s_{x,i}^{2} & s_{\text{xy},i}\\ s_{\text{xy},i} & s_{y,i}^{2} \end{array}\right)$ for all groups *i* = 1,19. Here${\bar{x}}_{i}$ and${\bar{y}}_{i}$ are, respectively, group means of expression levels of genes *x* and *y*, and$s_{x,i}^{2}$,$s_{y,i}^{2}$, and *s*_*xy*,*i*_ are group variances and covariances of expression levels of genes *x* and *y*, respectively.

2. Estimate asymptotic coefficients${\hat{\tau }}_{\text{xy}}$ as

(7) $${\hat{\tau }}_{\text{xy}}=\frac{{\bar{s}}_{\text{xy}}+d_{\text{xy}}}{d_{x}d_{y}}$$

where

(8) $${\bar{s}}_{\text{xy}}=\overset{19}{\underset{i=1}{\sum }}\lambda_{i}s_{\text{xy},i}$$

(9) $$d_{\text{xy}}=\overset{19}{\underset{i=1}{\sum }}\overset{19}{\underset{j>i}{\sum }}\lambda_{i}\lambda_{j}({\bar{x}}_{i}-{\bar{x}}_{j})({\bar{y}}_{i}-{\bar{y}}_{j})$$

(10) $$\begin{array}{ll} d_{x}^{2}=\bar{s_{x}^{2}}+\overset{19}{\underset{i=1}{\sum }}\overset{19}{\underset{j>i}{\sum }}\lambda_{i}\lambda_{j}{\left({\bar{x}}_{i}-{\bar{x}}_{j}\right)}^{2} & \; \end{array}$$

(11) $$\begin{array}{ll} d_{y}^{2}=\bar{s_{y}^{2}}+\overset{19}{\underset{i=1}{\sum }}\overset{19}{\underset{j>i}{\sum }}\lambda_{i}\lambda_{j}{\left({\bar{y}}_{i}-{\bar{y}}_{j}\right)}^{2} & \; \end{array}$$

(12) $$\begin{array}{ll} \bar{s_{x}^{2}}=\overset{19}{\underset{i=1}{\sum }}\lambda_{i}s_{x,i}^{2} & \; \end{array}$$

(13) $$\begin{array}{ll} \bar{s_{y}^{2}}=\overset{19}{\underset{i=1}{\sum }}\lambda_{i}s_{y,i}^{2} & \; \end{array}$$

3. Use the residual error ($r_{\text{xy}}-{\hat{\tau }}_{\text{xy}}$) to compare Pearson correlation coefficients as obtained from the large expression matrix and coefficients as estimated through Equation 7, which are based on parameter estimates of 19 groups of data.

4. Small residual errors indicate good agreement between *r*_*xy*_ and${\hat{\tau }}_{\text{xy}}$. As a result, the two components of Equation 7 (i.e. the weighted average of all covariances across 19 groups and the weighted average of the cross product of mean differences of a gene pair *xy* across 19 groups) can explain the signs and magnitudes of *r*_*xy*_, the Pearson correlation coefficient obtained from the large expression matrix.

### Pearson correlation coefficients obtained from the pooled expression data

We first obtained Pearson correlation coefficients for pairwise combinations of all 22,810 genes present in the large expression matrix, which resulted in more than 260 million coefficients. The Pearson correlation coefficients ranged from −0.992 to 0.998 with 0.008 as the mean value, and coefficients showed a symmetric distribution around zero; roughly 10% of these coefficients were greater than 0.7 or less than −0.7 (data shown in Additional file 1).

**Figure 4:**
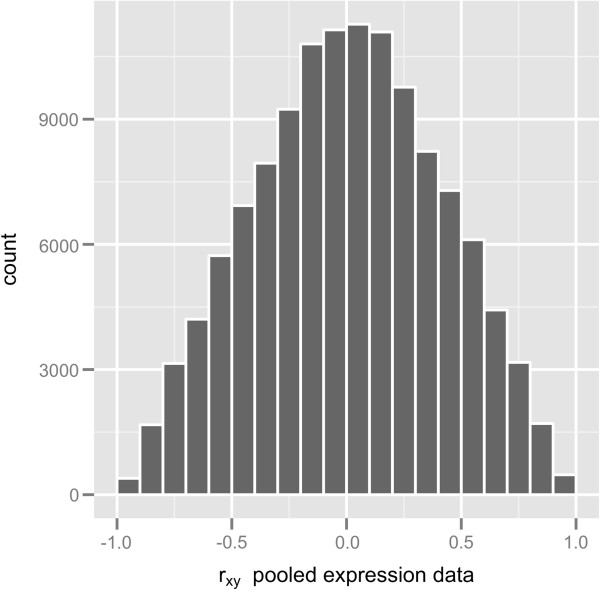
Histogram of 124,750 Pearson correlation coefficients obtained from the large expression matrix.

**Figure 5:**
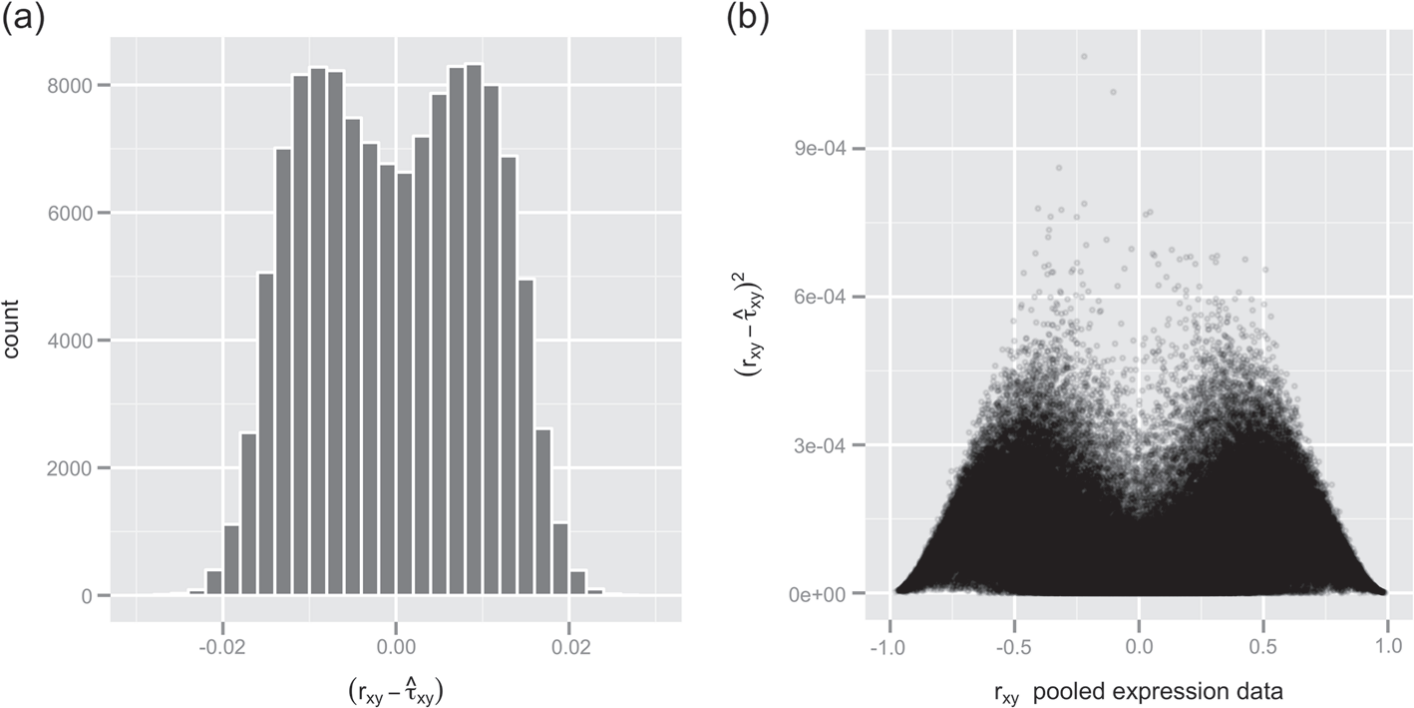
**Residual errors between *r***_***xy***_** and**${\hat{\tau }}_{\text{xy}}$**. (a)** Histogram of residual errors$\left(r_{\text{xy}}-{\hat{\tau }}_{\text{xy}}\right)$; **(b)** squared-residual errors${\left(r_{\text{xy}}-{\hat{\tau }}_{\text{xy}}\right)}^{2}$ vs. *r*_*xy*_.

Because all genes present in the large expression matrix provide more than 260 million pairwise correlation coefficients, we used a subset of 500 randomly selected genes and all their 124,750 pairwise correlation coefficients to illustrate potential problems with gene pairwise correlation coefficients estimated from a pool of microarray data. Pearson correlation coefficients from the pooled expression data (*r*_*xy*_) ranged from −0.979 to 0.990 with 0.007 as the mean value, and coefficients showed a symmetric distribution around zero; roughly 10% of these coefficients were greater than 0.7 or less than −0.7, as shown in the histogram of Figure 4. The asymptotic coefficients${\hat{\tau }}_{\text{xy}}$, estimated according to Equation 7, ranged from −0.978 to 0.989 with 0.007 as the mean value (${\hat{\tau }}_{\text{xy}}$ values were obtained through the R function given in Additional file 3). The histogram of residual errors$\left(r_{\text{xy}}-{\hat{\tau }}_{\text{xy}}\right)$ (Figure 5a) shows a bimodal distribution in which the mean value of negative residual errors is −0.008 and the mean value of positive residual errors is 0.008. The bimodality of the residual errors implies that$\left|r_{\text{xy}}|>|{\hat{\tau }}_{\text{xy}}\right|$. In addition, the plot of${\left(r_{\text{xy}}-{\hat{\tau }}_{\text{xy}}\right)}^{2}$ versus *r*_*xy*_ (Figure 5b) shows that residual errors are smaller closer to extreme values and reach a maximum around ±0.45. The bimodality observed in Figure 5a and the shape observed in Figure 5b closely follow the bimodality and shape of the bias between the Pearson estimator and the true correlation of a population *ρ*, which is approximately *ρ*(1−*ρ*^2^)/(2*n*) [32,33]. The bias *ρ*(1−*ρ*^2^)/(2*n*) is maximized as *ρ* assumes a value around ±0.58.

The analysis of all 124,750 pairwise correlations of 500 randomly selected genes revealed good agreement between *r*_*xy*_ and${\hat{\tau }}_{\text{xy}}$, despite the approximations we made about homogeneity of data within groups and the relatively low number of elements in each group; in our example data set 12 ≤ *n*_*i*_ ≤ 18, whereas Hassler and Thadewald’s example data set had around 90 elements in each of two groups [9]. Therefore, our analysis reassured us that the Pearson correlation coefficients obtained from the large expression matrix can be explained by heterogeneities due to different means and variances-covariances across the 19 groups we used to classify our example data set.

**Figure 6:**
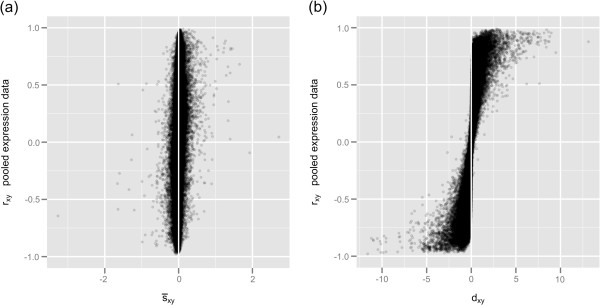
**Influence of covariances and means of 19 groups on signs of the Pearson correlation coefficients obtained from the pooled expression data. (a)**${\bar{s}}_{\text{xy}}=\sum_{i}^{19}\lambda_{i}s_{\text{xy},i}$; **(b)**$d_{\text{xy}}=\sum_{i=1}^{19}\sum_{j=i+1}^{19}\lambda_{i}\lambda_{j}({\bar{x}}_{i}-{\bar{x}}_{j})({\bar{y}}_{i}-{\bar{y}}_{j})$.

Next, we show the influence of each term of Equation 7 on signs and magnitudes of *r*_*xy*_, the Pearson correlation coefficients obtained from the large expression matrix. The plot of *r*_*xy*_ versus${\bar{s}}_{\text{xy}}$ (Figure 6a) shows that *r*_*xy*_ ranges from −1 to +1 for negative and positive values of${\bar{s}}_{\text{xy}}$. Therefore, positive or negative covariances of a gene pair within each of the 19 groups have no effect on positive or negative correlations estimated from the large expression matrix. Conversely, the “S” shape observed in plot of *r*_*xy*_ versus *d*_*xy*_ (Figure 6b) indicates that positive or negative mean-differences *d*_*xy*_ (Equation 9) of a gene pair across the 19 groups are the sole determinant of the sign of *r*_*xy*_, i.e. *d*_*xy*_ > 0 ⇒ *r*_*xy*_ > 0 and *d*_*xy*_ < 0 ⇒ *r*_*xy*_ < 0. The magnitude of *r*_*xy*_ is due mostly to mean differences because the correlation between *r*_*xy*_ and$\frac{d_{\text{xy}}}{d_{x}d_{y}}$ is 0.98 (the second term of Equation 7), whereas the correlation between *r*_*xy*_ and$\frac{{\bar{s}}_{\text{xy}}}{d_{x}d_{y}}$ is 0.31 (the first term of Equation 7).

Because |*r*_*xy*_| > 0.7 obtained from pools of microarray data has been used as the cut-off value representing a strong association between gene pairs [21-23], we computed the percentage contribution of the covariance and mean differences terms on the magnitude of |*r*_*xy*_| ≥ 0.7, i.e.$\frac{{\bar{s}}_{\text{xy}}}{r_{\text{xy}}d_{x}d_{y}}+\frac{d_{\text{xy}}}{r_{\text{xy}}d_{x}d_{y}}\approx 1$. There were 10,567 correlation coefficients with roughly equal numbers distributed in the *r*_*xy*_ < −0.7 and *r*_*xy*_ > 0.7 categories. The median of$\frac{{\bar{s}}_{\text{xy}}}{r_{\text{xy}}d_{x}d_{y}}\%$ was 1.98*%* with 50% of the data showing values between 0.04*%* and 5.32*%*. Conversely, the median of$\frac{d_{\text{xy}}}{r_{\text{xy}}d_{x}d_{y}}\%$ was 96.93*%* with 50% of the data showing values between 93.51*%* and 98.98*%*.

A combination of correlation coefficients between expression profiles within each group, given by${\bar{r}}_{\text{xy}}=\overset{19}{\underset{i=1}{\sum }}\lambda_{i}r_{\text{xy},i}$, ranged from$-0.6<{\bar{r}}_{\text{xy}}<0.89$ with 0.132 as the mean value. A direct comparison between *r*_*xy*_ and${\bar{r}}_{\text{xy}}$ showed a correlation coefficient of 0.3.

By applying the asymptotic theory developed by Hassler and Thadewald [9] to the Pearson correlation coefficients obtained from the large expression matrix, we showed that differences in means across 19 heterogeneous groups of data is the main factor determining the magnitude and sign of coefficients of 124,750 gene pairs. As previously shown by Hassler and Thadewald [9], this result corroborates that gene pairwise correlation coefficients estimated from a pool of microarray data do not measure “the closeness of linear relationship” [34] (p. 177) between expressions of a gene pair. Instead, they measure the extent of mean differences of a gene pair across different groups comprising the pool of data.

### Heteroskedasticity across a pool of microarray data

Here, we examine the case in which Pearson correlation coefficients are obtained from a pool of microarray data in which only gene pairwise variances-covariances differ across groups of data, i.e. Σ_*xy*,*i*_ ≠ Σ_*xy*,*j*_ for *i* ≠ *j*. In this case, Equation 1 can be written as

(14) $$r_{\text{xy}}^{*}\overset{p}{\to }\tau_{\text{xy}}^{*}=\frac{\overset{N}{\underset{i=1}{\sum }}\lambda_{i}\sigma_{\text{xy},i}}{\sqrt{\overset{N}{\underset{i=1}{\sum }}\lambda_{i}\sigma_{x,i}^{2}\overset{2}{\underset{i=1}{\sum }}\lambda_{i}\sigma_{y,i}^{2}}}$$

For instance, heteroskedasticity could occur in a situation in which data from completely replicated microarray experiments are pooled to be examined as one data set. As was reported in the work of Goldstein *et al.*[1], data variability could differ substantially across replicated microarray experiments.

In order to attain only heteroskedasticity across the 19 groups of our example data set, we removed the effect of varied experimental conditions on expressions of genes within each group. For this purpose, we fitted linear models to genes (within each group *i* = 1,19) and obtained their residuals. Following the methodology for differential expression of genes proposed by Smith [35], we modeled the expression level of all genes in group *i*, here represented by a matrix *Y*_*i*_, with a systematic treatment effect (a linear model represented by *Z*_*i*_*β*_*i*_) plus error, i.e.

$$Y_{i}=Z_{i}\beta_{i}+\varepsilon_{i}$$

for *i* = 1,19. We assumed that *ε*_*i*_∼*N*(0,Σ_*i*_), where Σ_*i*_ is the variance-covariance matrix of all genes in each group *i* = 1,19. We obtained residuals as

$${\hat{\varepsilon }}_{i}=Y_{i}-Z_{i}{\hat{\beta }}_{i}$$

where${\hat{\beta }}_{i}$ was estimated using the open-source Bioconductor R package Limma [30,31]. This approach is equivalent to subtracting expression levels of each biological replicate from their mean values. We used linear models because they are well known by the community who works with differential expression of microarrays measurements and the process of obtaining their residuals is easy and automatic through the use of the Limma package [30,31].

We combined all gene expression residuals from the 19 groups into one pool of residuals (a large matrix of residuals including 520 columns and 22,810 rows). Expression levels of the two treatment conditions “genotoxic stress applied to root 1 hour post-treatment” and “heat control applied to shoot 24 hours post-treatment” could not be used in the analysis of residuals because they had only one biological replicate (refer to Additional file 2 for more details). This explains why the matrix of residuals has 520 columns instead of 522 columns. We repeated the analysis described in steps 1–4 from the section “Application to experimental microarray data” for the data in the large matrix of residuals.

### Pearson correlation coefficients estimated from the pooled residuals

Here we show results of the analysis involving the large matrix of residuals (pooled residuals) for the same subset of 500 genes used in the analysis of the large expression matrix. Pearson correlation coefficients ($r_{\text{xy}}^{*}$) of all 124, 750 pairwise combinations of 500 genes obtained from the large matrix of residuals ranged from −0.553 to 0.849 with 0.01 as the mean value. Their asymptotic counterparts (${\hat{\tau }}_{\text{xy}}^{*}$), estimated according to Equation 7, ranged from −0.554 to 0.849 with 0.01 as the mean value. The combination of covariances within each of the 19 groups, i.e.$\overset{19}{\underset{i=1}{\sum }}\lambda_{i}s_{\text{xy},i}$, determined the sign of$r_{\text{xy}}^{*}$ because all pairwise mean differences among groups were zero (data shown in Additional file 1).

We then compared$r_{\text{xy}}^{*}\approx \frac{\overset{19}{\underset{i=1}{\sum }}\lambda_{i}s_{\text{xy},i}}{\sqrt{\overset{19}{\underset{i=1}{\sum }}\lambda_{i}s_{x,i}^{2}\overset{19}{\underset{i=1}{\sum }}\lambda_{i}s_{y,i}^{2}}}$ to the weighted average of correlations obtained within each of the 19 groups of residuals, i.e.${\bar{r}}_{\text{xy}}^{*}=\overset{19}{\underset{i=1}{\sum }}\lambda_{i}r_{\text{xy},i}^{*}$;${\bar{r}}_{\text{xy}}^{*}$ ranged from −0.631 to 0.847 with 0.011 as the mean value. In addition, we observed a strong linear relationship between$r_{\text{xy}}^{*}$ estimated from the large matrix of residuals and${\bar{r}}_{\text{xy}}^{*}$, with a correlation equal to 0.93. Therefore, the Pearson correlation coefficients obtained from the large matrix of residuals (whose heterogeneities result from different variances-covariances across the 19 groups) also measure a linear relationship between the expression residuals of a gene pair.

### Bias of correlation coefficients obtained across 19 groups of microarray data

We provide here a performance metric for the correlation coefficients estimated across the 19 groups of microarray data by assessing their bias with respect to coefficients within each of the 19 groups. We quantified bias as in Equation 15:

(15) $$B({\hat{\rho }}_{\text{xy}})=\sqrt{\frac{\overset{19}{\underset{i=1}{\sum }}\lambda_{i}{\left({\hat{\rho }}_{\text{xy}}-{\hat{\rho }}_{\text{xy},i}\right)}^{2}}{19}}$$

where${\hat{\rho }}_{\text{xy}}$ represents the correlation point estimate of a gene pair *xy* across the 19 groups of microarray data and${\hat{\rho }}_{\text{xy},i}$ represents its counterparts within each group.

We evaluated the bias (as defined in Equation 15) of each of the 124,750 gene pairs’ correlation coefficients that were obtained according to: (a)${\hat{\rho }}_{\text{xy}}=r_{\text{xy}}$, the Pearson correlation coefficients obtained directly from the large expression matrix (pooled data); (b)${\hat{\rho }}_{\text{xy}}={\bar{r}}_{\text{xy}}$, the average of correlations between expression profiles within *i* = 1,19 groups comprising the large expression matrix; (c)${\hat{\rho }}_{\text{xy}}=r_{\text{xy}}^{*}$, the Pearson correlation coefficients estimated directly from the large matrix of residuals (pooled residuals); and (d)${\hat{\rho }}_{\text{xy}}={\bar{r}}_{\text{xy}}^{*}$, the average of correlations between expression residuals within *i* = 1,19 groups comprising the large matrix of residuals. For the large expression matrix,${\hat{\rho }}_{\text{xy},i}=r_{\text{xy},i}$ is the Pearson correlation coefficient between expression profiles within each of 19 groups comprising the large expression matrix, whereas for the large matrix of residuals,${\hat{\rho }}_{\text{xy},i}=r_{\text{xy},i}^{*}$ is the Pearson correlation coefficient between expression residuals within each of 19 groups comprising the large matrix of residuals. Table 2 gives the statistical summaries of the values obtained for *B*(*r*_*xy*_),$B({\bar{r}}_{\text{xy}})$,$B(r_{\text{xy}}^{*})$, and$B({\bar{r}}_{\text{xy}}^{*})$.

**Table 2:** Statistical summaries of biases of correlation coefficients

	**Min.**	**1st Qu.**	**Median**	***3rdQu.***	***Max.***
*B*(*r*_*xy*_)	0.022	0.078	0.099	0.132	0.308
$B({\bar{r}}_{\text{xy}})$	0.021	0.062	0.072	0.083	0.153
$B(r_{\text{xy}}^{*})$	0.019	0.049	0.057	0.067	0.141
$B({\bar{r}}_{\text{xy}}^{*})$	0.018	0.049	0.057	0.065	0.132

***B(r***_***xy***_***)*** – bias of the Pearson correlation coefficients estimated directly from the large expression matrix;$B({\bar{r}}_{\text{xy}})$ – bias of the average of correlations between expression profiles within ***i = 1,19*** groups; ***B(rxy ∗)*** – bias of the Pearson correlation coefficients estimated directly from the pooled residuals;$B({\bar{r}}_{\text{xy}}^{*})$ – bias of the average of correlations between expression residuals within ***i = 1,19*** groups.

The analysis involving the data in the large expression matrix (whose heterogeneities were due to means and variances-covariances differences across 19 groups) resulted in consistently larger statistical summaries of *B*(*r*_*xy*_) than did those of$B({\bar{r}}_{\text{xy}})$. In addition, the maximum value of *B*(*r*_*xy*_) is twice as much the maximum value of$B({\bar{r}}_{\text{xy}})$ (Table 2). For the large matrix of residuals (whose heterogeneities were due only to heteroskedasticity), the values of$B(r_{\text{xy}}^{*})$ shown in Table 2 are slightly larger than are the values of$B({\bar{r}}_{\text{xy}}^{*})$.

**Figure 7:**
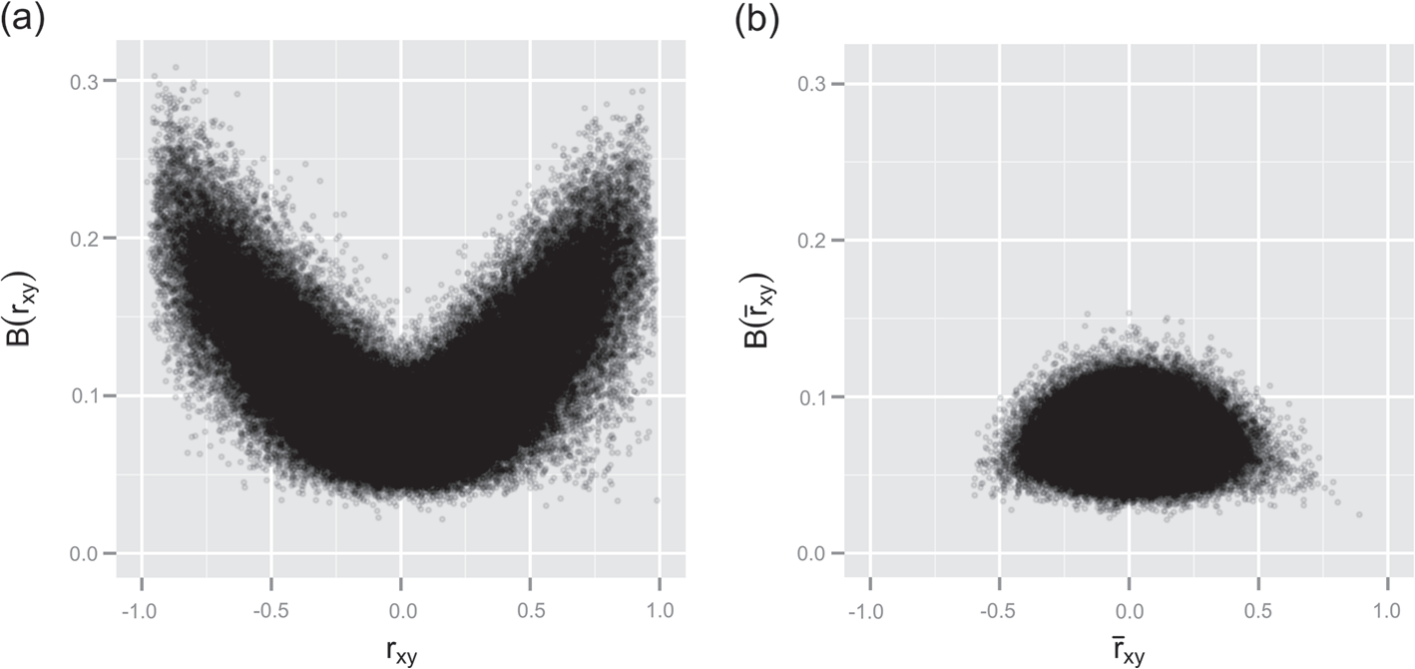
**Assessment of biases of the correlation coefficients estimated from 19 groups of expression data.**$B\left({\hat{\rho }}_{\text{xy}}\right)=\sqrt{\frac{\overset{19}{\underset{i=1}{\sum }}\lambda_{i}{\left({\hat{\rho }}_{\text{xy}}-{\hat{\rho }}_{\text{xy},i}\right)}^{2}}{19}}$ for **(a)**${\hat{\rho }}_{\text{xy}}=r_{\text{xy}}$, the Pearson correlation coefficients estimated directly from the large expression matrix; **(b)**${\hat{\rho }}_{\text{xy}}={\bar{r}}_{\text{xy}}$, the average of correlations between expression profiles within *i* = 1,19 groups;${\hat{\rho }}_{\text{xy},i}$ is the correlation between expression profiles within each group.

Moreover, more information can be grasped through the visualization of biases versus coefficients, as shown in Figures 7a and 7b. The trend shown in the plot of *B*(*r*_*xy*_) versus *r*_*xy*_ (Figure 7a), where *B*(*r*_*xy*_) increases as |*r*_*xy*_| approaches ±1, is very distinct from that shown in the plot of *B*(*r*_*xy*_) versus${\bar{r}}_{\text{xy}}$ (Figure 7b), where *B*(*r*_*xy*_) decreases as${\bar{r}}_{\text{xy}}$ approaches ±1. Indeed, the mean value *B*(*r*_*xy*_) for |*r*_*xy*_| > 0.7 is 0.18, whereas the mean value of$B({\bar{r}}_{\text{xy}})$ for$|{\bar{r}}_{\text{xy}}|>0.7$ is 0.045. The visualization of biases involving the large matrix of residuals showed a roughly random pattern between$B(r_{\text{xy}}^{*})$ and$\left|r_{\text{xy}}^{*}\right|$, as$\left|r_{\text{xy}}^{*}\right|$ decreases to zero (data shown in Additional file 1). The plot of$B({\bar{r}}_{\text{xy}}^{*})$ versus${\bar{r}}_{\text{xy}}^{*}$ showed a pattern similar to the one observed in Figure 7b, where$B({\bar{r}}_{\text{xy}}^{*})$ decreases as${\bar{r}}_{\text{xy}}^{*}$ approaches ±1 and both$B({\bar{r}}_{\text{xy}}^{*})$,$B({\bar{r}}_{\text{xy}})$ show maximum values around zero (data shown in Additional file 1).

**Figure 8:**
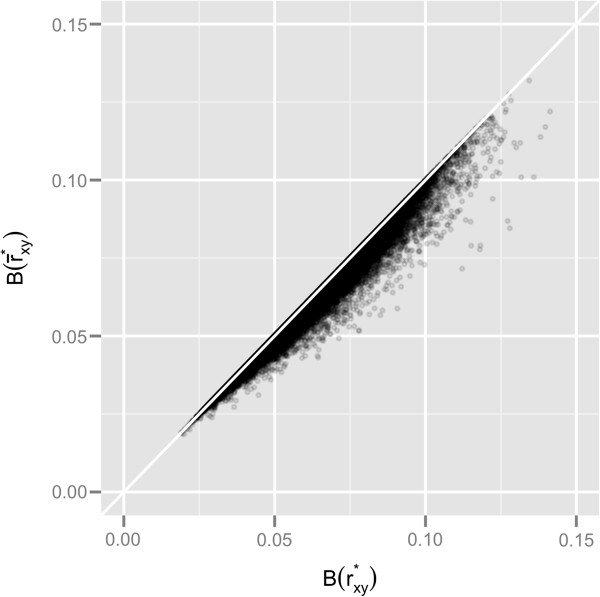
**Comparison between**$B(r_{\text{xy}}^{*})$ and$B({\bar{r}}_{\text{xy}}^{*})$**.**$B(r_{\text{xy}}^{*})$ is the bias of the Pearson correlation coefficients estimated directly from the pooled residuals;$B({\bar{r}}_{\text{xy}}^{*})$ is the bias of the average of correlations between expression residuals within *i* = 1,19 groups.

The plot of$B({\bar{r}}_{\text{xy}}^{*})$ versus$B(r_{\text{xy}}^{*})$ (Figure 8) reveals all data points lying below the diagonal, thus implying that$B({\bar{r}}_{\text{xy}}^{*})<B(r_{\text{xy}}^{*})$**, ∀**${\bar{r}}_{\text{xy}}^{*}$,$r_{\text{xy}}^{*}$. This result corroborates that, in the case of only heteroskedasticity across the 19 groups of microarray data, the combination of correlation coefficients performs better than pooling data.

## Discussion and conclusion

### Discussion

In this study, we performed a comprehensive analysis of Pearson correlation coefficients obtained from combining data of 19 heterogeneous groups of experimental microarray data into one large pool. By applying the theory developed by Hassler and Thadewald [9] to our example data set, we determined the specific effect of mean differences and heteroskedasticity across the 19 groups on the sign and magnitude of the pooled coefficients. In addition, we provided a performance metric for correlation coefficients by quantifying their biases.

We quantified the bias of the correlation coefficient of a gene pair through the mean squared error between its estimate across a pool of groups and its estimates within groups. A similar method has been used by Hunter and Schmidt to assess the variance of their meta-analysis estimator of the Pearson correlation coefficient across independent studies [36]. We evaluated the bias of gene-to-gene correlations estimated according to the following two methods: (a) by combining 19 groups of microarray data into a large pool to be analyzed as a single data set (pooled data) and (b) by combining correlation coefficients of each of 19 groups of microarray data into an average weighted by the number of elements in each group, which corresponds to the Hunter-Schmidt meta-analysis estimator of the Pearson correlation coefficient across independent studies [36].

The data used in this study came from 10 microarray experiments (AtGenExpress Project [27]) carried out by seven different laboratories distributed across Germany that followed the same experimental protocol; these are a subset of the large pool of microarray data found in the study of Horan *et al.*[22]. Experiments followed a 3-factorial design with treatment (abiotic stress, control), tissue (root, shoot, or seedlings in general), and time post-treatment as factors [27]. Mean differences within and across experiments were a matter-of-fact because statistically significant differences in gene expression of several types of abiotic stress versus control treatment were reported in Kilian *et al.*’s study [27]. We expected differences due to variability across experiments to remain after removing mean differences because of reported difficulties in the reproduction of microarray studies [1]. Therefore, homogeneity cannot be ensured across experiments, and combining the means or residuals of biological replicates of the 10 experiments into a large pool as a single set is not sound from a statistical viewpoint.

The analysis of the components of the correlation coefficients obtained from the large expression matrix corroborated the results predicted by both theory and simulation that variances-covariances within the 19 groups had negligible impact on correlation coefficients, but different means across the 19 groups had a decisive effect on the sign as well as on the magnitude of coefficients. Coefficients that were greater than 0.7 or less than −0.7 showed the largest range of bias (Table 2). Therefore, large values of the pooled coefficients were an artifact in the sense that they did not communicate a real linear association between the expression profiles of two genes; rather, they appeared because the data were combined into a large pool. For this reason, large values of the pooled coefficients are in fact an ecological fallacy [10].

We also showed through Monte Carlo simulation that the structure of different means across a pool of 10 ≤ *N* ≤ 100 groups could generate Simpson’s paradox. In our case study simulation shown in Figure 2, we showed that even though the correlation within each group was +0.9, a pool of *N* (10 ≤ *N* ≤ 100) groups provided negative correlation coefficients because the combination of all pairwise mean differences had a negative sign and greater magnitude than the positive covariance of the data within groups. Hassler and Thadewald [9] studied Simpson’s paradox through the analytical solution of Equation 1 for *N* = 2, and showed that the occurrence of mean differences with opposite signs in both correlated variables is a condition for contradictory results between a correlation coefficient that is estimated across or within each of two groups.

We combined residuals from fitting linear models of every gene into a large matrix of residuals (22,810 rows x 520 columns). Here we departed from the assumption of independence (common to the analysis of differentially expressed genes [35,37]) and considered a multivariate normal distribution for residuals within groups, with a mean of zero and variance-covariance Σ_*xy*,*i*_, *i* = 1,19. The large matrix of residuals gave us the opportunity to evaluate gene pair correlations estimated from a pool of data marked by only heteroskedasticity. Our results showed that correlation coefficients estimated across the 19 groups of residuals were closely related to the variance-covariances within groups. We also found a strong linear relationship between the Pearson correlation coefficients obtained from the large matrix of residuals and the coefficients resulting from averaging correlation estimates within groups. However, the heteroskedasticity of the data in the large matrix of residuals resulted in less efficient estimations of the correlation between a gene pair than did the classical meta-analysis approach of combining correlation coefficients into an average. These results were corroborated by Monte Carlo simulations of only heteroskedasticity across *N* > 2 groups of data.

The results shown in this study indicate that the combination of statistical results is best suited for estimating correlations of a gene pair across several microarray studies. Nevertheless, further studies are necessary to assess various methods of combining within-groups gene-to-gene correlation coefficients.

### Conclusion

This study demonstrates three aspects of the importance of statistical methods in the synthesis of information across microarray experiments: 

(A) Large values of gene-to-gene Pearson correlation coefficients estimated from a pool of 19 groups of microarray data were an ecological fallacy; the effect of heterogeneous means across a pool of data overpowers the covariance structure of the data.

(B) The effect of heterogeneous variance-covariances across a pool of data causes less efficient estimates of Pearson correlation coefficients across groups of microarray data than does the approach of combining correlation coefficients of individual groups.

(C) The combination of statistical results is best suited for synthesizing the correlation between expression profiles of a gene pair across several microarray studies.

## Competing interests

The authors declare that they have no competing interests.

## Authors’ contributions

MMAM elaborated the project idea, compiled the example data set, performed the statistical analysis, and wrote major parts of the paper. NR downloaded the cel files from TAIR. YF and JH contributed to the project idea and editing of the manuscript. ESW wrote parts of the paper and provided the biological input. All authors read and approved the final manuscript.

## Supplementary Material

Additional file 1 This file contains additional figures detailing results. Click here for file

Additional file 2 This file gives detailed information about treatment conditions and number of biological replicates in each of the 10 microarray experiments of our example data set. Click here for file

Additional file 3 **This file gives the R function that estimates**${\hat{\tau }}_{\text{xy}}$**,**${\bar{s}}_{\text{xy}}$**,** ***d***_***xy***_**,** ***d***_***x***_** and *d***_***y***_. Click here for file
