# Supplementary material for: Comprehensive analysis of correlation coefficients estimated from pooling heterogeneous microarray data
Source: BMC Bioinformatics. 2013 Jul 4;14:214. doi: 10.1186/1471-2105-14-214 (PMC3765419; doi:10.1186/1471-2105-14-214)
Supplement: Additional file 1 — This file contains additional figures detailing results. [file 1471-2105-14-214-S1.pdf]

Supplemental Figures

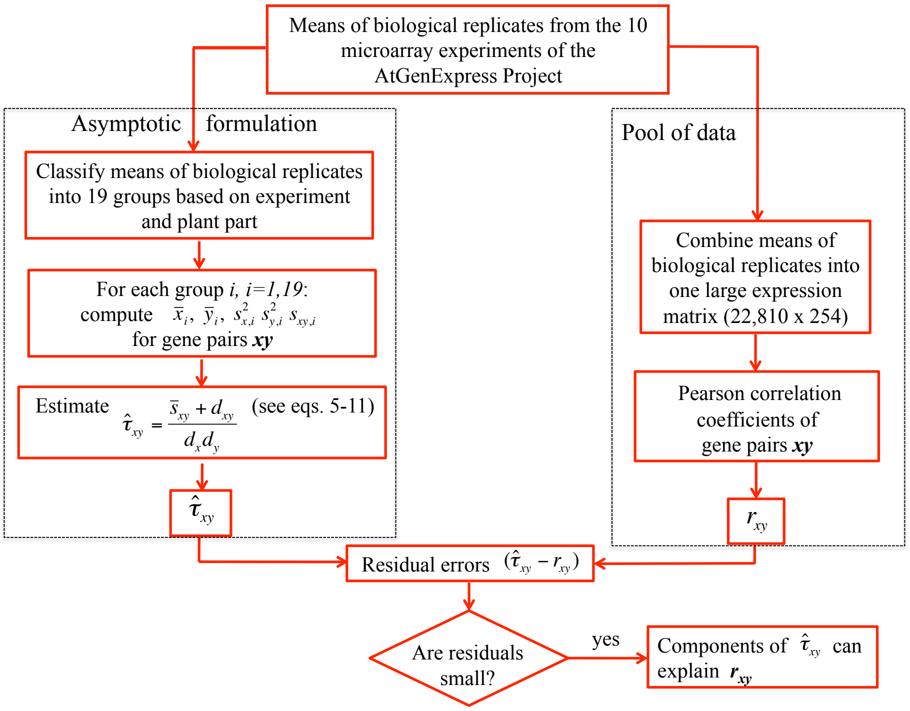

Figure 1: Diagram shows an overview of our methodology for dissecting the components of the Pearson correlation coefficient obtained r_{xy} from a pool of 19 groups of microarray data.

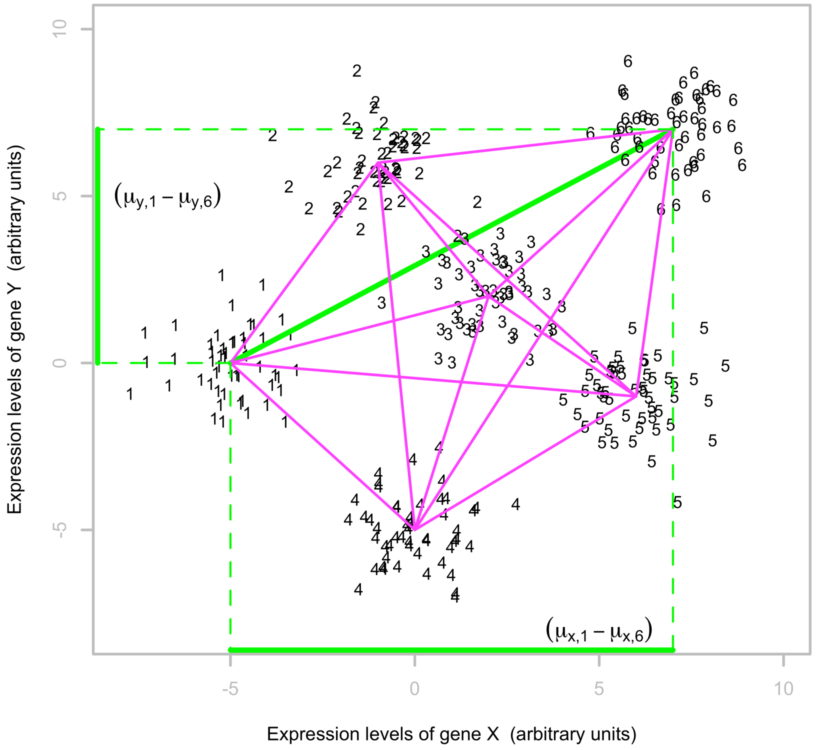

Figure 2: Illustration in a two-dimensional gene expression space of the effect of heterogeneities due to groups' mean differences on Pearson correlation coefficients: combination of 6 groups into a pool; each group contains 50 data points representing a gene-pair xy simulated according to multivariate normal distributions, where $\Sigma_{xy,i} = \begin{pmatrix} 1 & 0 \\ 0 & 1 \end{pmatrix}$ for i=1,6 and $\mu_{xy,i} \neq \mu_{xy,j}$ for $i \neq j$. Data points of each group are portrayed by their corresponding group number in a two-dimensional gene expression space. Segments represent vectors of differences between means among all pairwise combinations of 6 groups. Projections into gene-X and gene-Y axes of the mean difference vector between groups 1 and 6 are shown as a green segment. The sum of all cross products between pairs of projections has a determining effect on Pearson correlations estimated directly from the pool of groups.

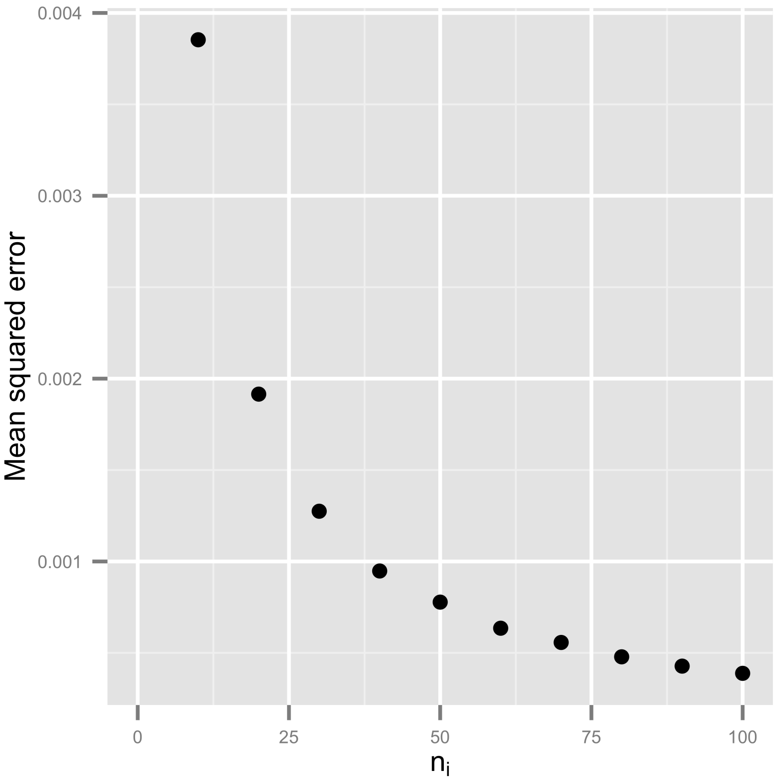

Figure 3: Mean-squared error $(\tau_{xy} - \hat{\tau}_{xy})^2$ vs. n_i $(10 \leq n_i \leq 100)$ shows the influence of number of elements within groups on estimates of the asymptotic coefficients. τ_{xy} was obtained from plugging population parameters $\mu_{xy,i}$ and $\Sigma_{xy,i}$ into equation 1, whereas $\hat{\tau}_{xy}$ was obtained from group parameters. The correspondence between τ_{xy} and $\hat{\tau}_{xy}$ is good even for $n_i = 10$.

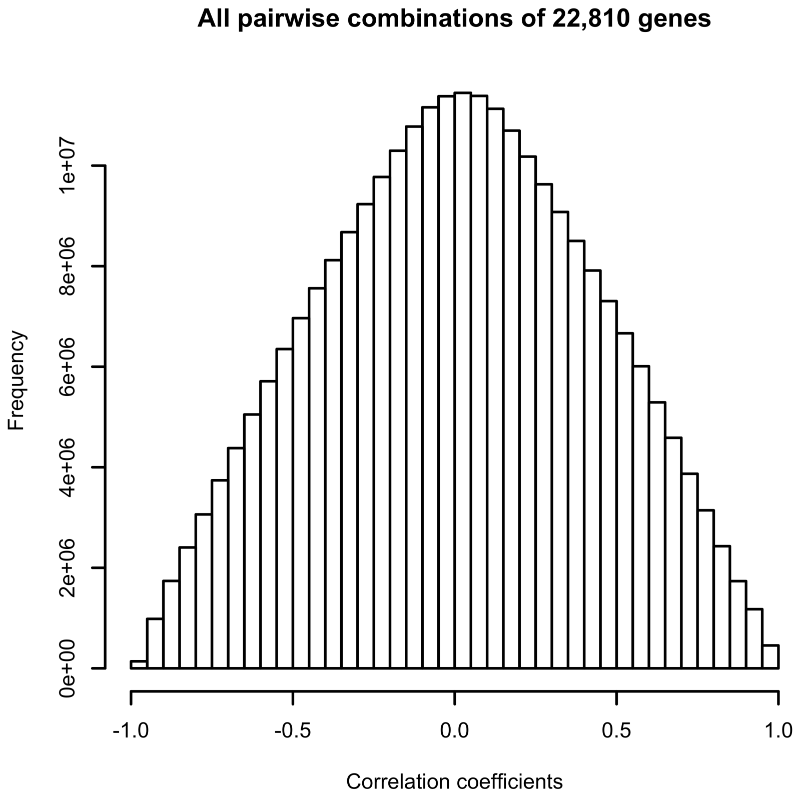

Figure 4: Histogram of Pearson correlation coefficients of all pairwise combinations of 22,810 genes (> 260 million coefficients) in the large expression matrix.

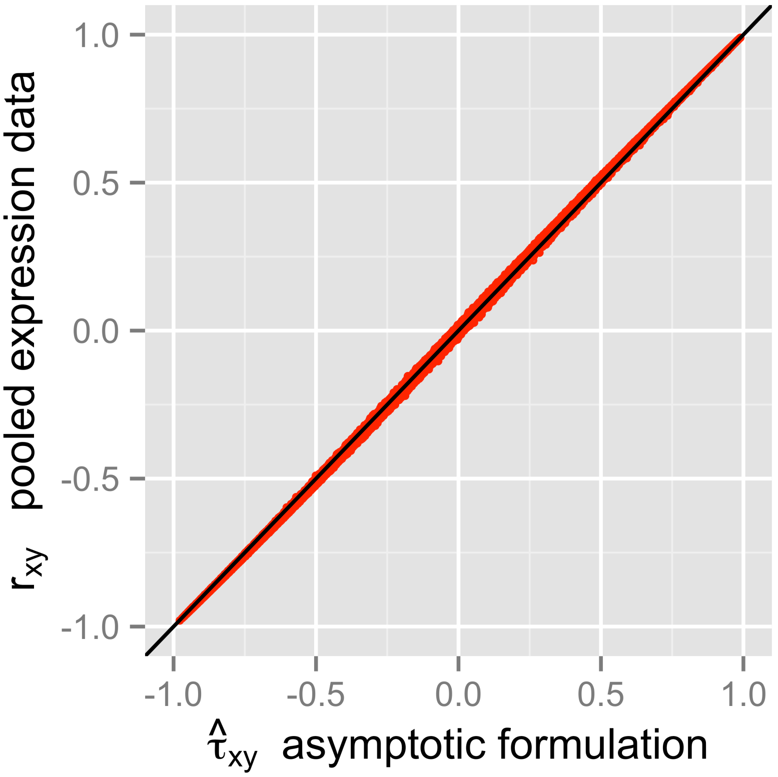

Figure 5: This scatterplot shows the good agreement between r_{xy} and $\hat{\tau}_{xy}$, in which data points lie around the diagonal. This plot also shows that data points tend to lie closer to the diagonal around ± 1, which indicates that residual errors $(r_{xy} - \hat{\tau}_{xy})$ tend to be smaller as $|r_{xy}|$ approaches ± 1. Interestingly, coefficients greater than 0.5 tend to show slightly larger values when estimated directly from the large expression matrix, whereas the contrary is observed for coefficients less than -0.5 (i.e. data points tend to lie above the diagonal for $r_{xy} > 0.5$, whereas they lie below the diagonal for $r_{xy} < -0.5$).

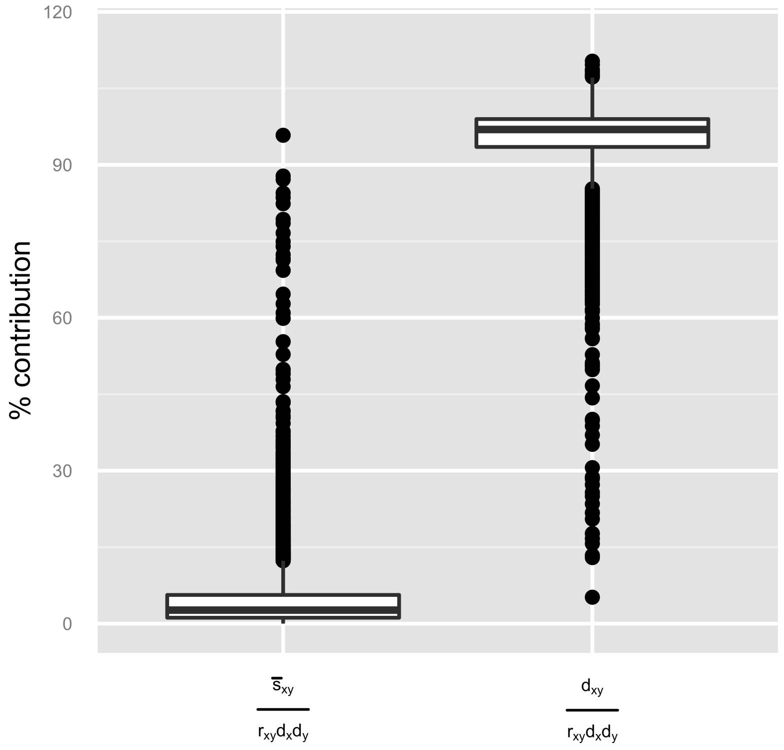

Figure 6: Boxplots of the percentage contribution of the covariance and the mean differences terms on the magnitude of $|r_{xy}| \geq 0.7$, i.e. $\frac{\bar{s}_{xy}}{r_{xy}d_xd_y} + \frac{d_{xy}}{r_{xy}d_xd_y} \approx 1$. The median of $\frac{\bar{s}_{xy}}{r_{xy}d_xd_y}\%$ is 1.98% with 50% of the data having a value between 0.04% and 5.32%. Conversely, the median of $\frac{d_{xy}}{r_{xy}d_xd_y}\%$ is 96.93% with 50% of the data having a value between 93.51% and 98.98%. There are 738 outliers in the right boxplot of Figure 7, ranging from 12% to 96%, but only 14 of them represent a contribution larger than 70% (the median of these outliers is 17%). There are 608 outliers in the right boxplot of Figure 7 ranging from 5% to 82%, and the median of these outliers is 80%. In addition, this boxplot shows 14 outliers whose contribution is above 100%, which then implies a negative contribution of the covariance term on the magnitude of r_{xy}.

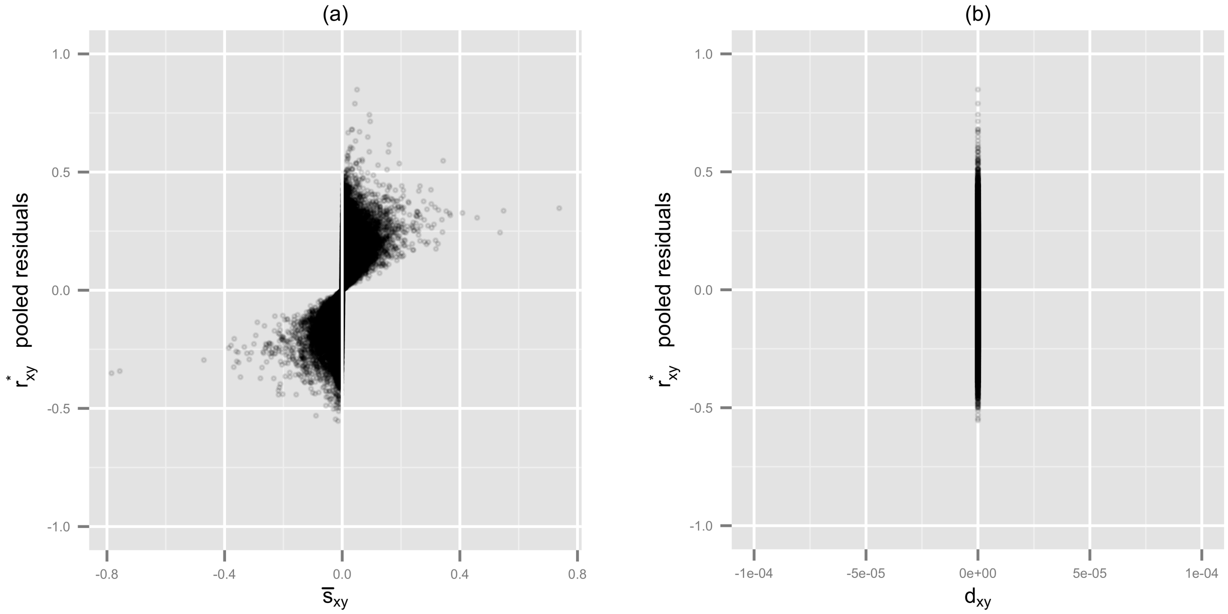

Figure 7: Influence of sample covariances and means of 19 groups on signs of Pearson correlation coefficients estimated from the pooled residuals: (a) r^*_{xy} vs. $\bar{s}_{xy} = \sum_i \lambda_i s_{xy,i}$ and (b) r^*_{xy} vs. $d_{xy} = \sum_i \sum_{j>i}^{19} \lambda_i \lambda_j (\bar{x}_i - \bar{x}_j)(\bar{y}_j - \bar{y}_j)$; r^*_{xy} have only the covariance component of equation 7 because mean differences among groups were successfully removed by fitting linear models to each gene.

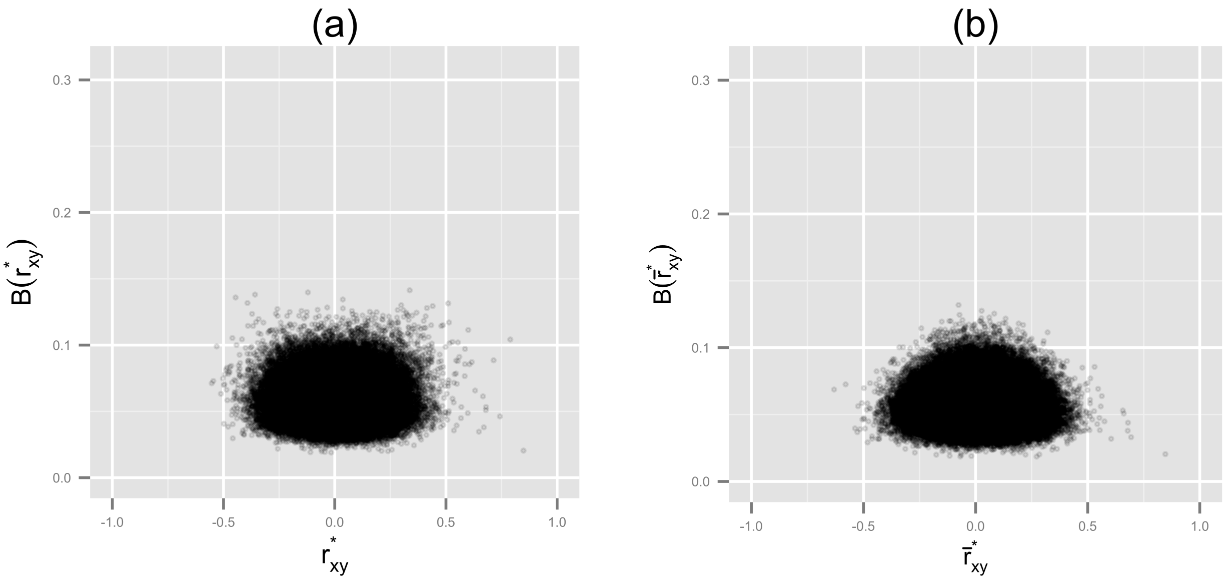

Figure 8: Assessment of biases of the correlation coefficients estimated from 19 groups of residuals data: $B(\hat{\rho}_{xy}) = \sqrt{\frac{\sum_{i=1}^{19} \lambda_i (\hat{\rho}_{xy} - \hat{\rho}_{xy,i})^2}{19}}$ for (a) $\hat{\rho}_{xy} = r^*_{xy}$, the Pearson correlation coefficients estimated directly from the pooled residuals; (b) $\hat{\rho}_{xy} = \bar{r}^*_{xy}$, the average of correlations between expression residuals within $i = 1, 19$ groups; $\hat{\rho}_{xy,i}$ is the correlation between expression residuals within each group.
