# Supplementary material for: Comprehensive analysis of correlation coefficients estimated from pooling heterogeneous microarray data
Source: BMC Bioinformatics. 2013 Jul 4;14:214. doi: 10.1186/1471-2105-14-214 (PMC3765419; doi:10.1186/1471-2105-14-214)
Supplement: Additional file 3 — This file gives the R function that estimatesτ^xy,s¯xy, dxy, dx and dy. [file 1471-2105-14-214-S3.rtf]

Group.Bias.fn<-function(n.chips,treatments,matriz.genexp,matriz.mean.group,matriz.sd.group){#n.chips -- number of chips per group#matriz.genexp -- this function require a matrix of gene expression, genes in columns and conditions in rows;#matriz.mean.group, matriz.sd.group -- this function requires means and standard deviations of each group that is considered homogeneous##sets group names in same order as group names of matrices of means and sdsgroup.names<-matriz.mean.group[,1]#takes group names out of first column of matrices of means and sdsmatriz.mean.group<-matriz.mean.group[,-1]matriz.sd.group<-matriz.sd.group[,-1]#====================================================#n1<-ncol(matriz.genexp)-2n2<-ncol(matriz.genexp)nome.first<-group.names[1]group.names<-group.names[-1]Cor.bias<-numeric(0); Cov.xy<-numeric(0); Shift.means<-numeric(0); Delta.xy<-numeric(0)ProbeID.number<-rep(0,2)#for (k in 1:n1){#=========================================================================================#Computes vector of correlations of each group, vetor.cor.groups for first pair of genes#=========================================================================================x<-matriz.genexp[treatments==nome.first,c(k,k+1)]vetor.cor.groups<-cor(x[,1],x[,2])for (i in group.names){	x<-matriz.genexp[treatments==i,c(k,k+1)]	vetor.cor.groups<-append(vetor.cor.groups,cor(x[,1],x[,2]))}	ProbeID.number<-rbind(ProbeID.number,c(k,k+1))	#cat(k,k+1,"\n")#==================================================================#Computes bias involving pair of genes k and k+1#==================================================================x<-Bias.cor.fn(matriz.mean.group[,k],matriz.mean.group[,k+1],matriz.sd.group[,k],matriz.sd.group[,k+1],n.chips,vetor.cor.groups)Cor.bias<-append(Cor.bias,x$Bias); Cov.xy<-append(Cov.xy,x$cov.xy) Shift.means<-append(Shift.means,x$shift.mean); Delta.xy<-append(Delta.xy,x$delta.XY)##==========================================================#Computes bias of all other pairs of genes#==========================================================for (j in (k+2):n2){#==========================================#Computes vector of correlations#==========================================	x<-matriz.genexp[treatments==nome.first,c(k,j)]	vetor.cor.groups<-cor(x[,1],x[,2])#		for (i in group.names){		x<-matriz.genexp[treatments==i,c(k,j)]		vetor.cor.groups<-append(vetor.cor.groups,cor(x[,1],x[,2]))}	ProbeID.number<-rbind(ProbeID.number,c(k,j))	#cat(k,j,"\n")#=====================================================================#Computes bias involving pair of genes k and j#=====================================================================		x<-Bias.cor.fn(matriz.mean.group[,k],matriz.mean.group[,j],matriz.sd.group[,k],matriz.sd.group[,j],n.chips,vetor.cor.groups)		Cor.bias<-append(Cor.bias,x$Bias); Cov.xy<-append(Cov.xy,x$cov.xy) 		Shift.means<-append(Shift.means,x$shift.mean); Delta.xy<-append(Delta.xy,x$delta.XY)	}}#======================================================k<-n2-1x<-matriz.genexp[treatments==nome.first,c(k,k+1)]vetor.cor.groups<-cor(x[,1],x[,2])	for (i in group.names){		x<-matriz.genexp[treatments==i,c(k,k+1)]		vetor.cor.groups<-append(vetor.cor.groups,cor(x[,1],x[,2]))}ProbeID.number<-rbind(ProbeID.number,c(k,k+1))	#cat(k,k+1,"\n")x<-Bias.cor.fn(matriz.mean.group[,k],matriz.mean.group[,k+1],matriz.sd.group[,k],matriz.sd.group[,k+1],n.chips,vetor.cor.groups)Cor.bias<-append(Cor.bias,x$Bias); Cov.xy<-append(Cov.xy,x$cov.xy) Shift.means<-append(Shift.means,x$shift.mean); Delta.xy<-append(Delta.xy,x$delta.XY)#ProbeID.number<-ProbeID.number[-1,]#invisible(data.frame(ProbeID.number,Cor.bias,Cov.xy,Shift.means,Delta.xy))}#==========================Function Group.Bias.fn ends here===================================##====================================================================================================#Function to compute bias of correlations from pooling heterogeneous groups #====================================================================================================Bias.cor.fn<-function(x.mean,y.mean,x.sd,y.sd,n.vetor,r1){#==================================================================#this version requires two vectors with mean of X and mean of Y,#two more vectors with sd of X and sd of Y, and #a vector of correlations between X and Y inside each of n groups#==================================================================	N.total<-sum(n.vetor)	n<-length(n.vetor)#=====================================#compute bias due to mean differences#=====================================shift.mean<-0delta.X<-0delta.Y<-0for (i in 2:n){	for(j in 1:(i-1)){	shift.mean<-shift.mean + 	(n.vetor[i]/N.total)*(n.vetor[j]/N.total)*(x.mean[i]-x.mean[j])*(y.mean[i]-y.mean[j])	#	delta.X<-delta.X + (n.vetor[i]/N.total)*(n.vetor[j]/N.total)*(x.mean[i]-x.mean[j])^2	delta.Y<-delta.Y + (n.vetor[i]/N.total)*(n.vetor[j]/N.total)*(y.mean[i]-y.mean[j])^2	}	#}#====================================================#Compute bias due to variance differences#====================================================	delta.X<-sum((n.vetor*x.sd^2)/N.total) + delta.X	delta.Y<-sum((n.vetor*y.sd^2)/N.total)+ delta.Y	delta.XY<-sqrt(delta.X*delta.Y)		#	#cov.xy<-sum((n.vetor*x.sd*y.sd)/N.total)#====================================================#compute bias due to covarainces differences#====================================================	cov.xy<-sum((n.vetor*x.sd*y.sd*r1)/N.total)	Bias<-(cov.xy+shift.mean)/delta.XY	resultados<-list()	resultados$cov.xy<-cov.xy	resultados$shift.mean<-shift.mean	resultados$delta.XY<-delta.XY	resultados$Bias<-Bias	return(resultados)}
